# 

**Published:** 1888-08

**Authors:** 


WHOLE NUMBER, CCCXXV.

August, 1888.

No. 1. VOL. XXVIII. New Series.

LIBRARY
INDEXED
S.G.O.

### BUFFALO MEDICAL AND SURGICAL JOURNAL.

Established 1845 by AUSTIN FLINT, M. D.

PUBLISHED MONTHLY.

Editors:

THOS. LOTHROP, M. D. W. W. POTTER, M. D.

Associate Editors:

FLOYD S. CREGO, M. D. FRED. R. CAMPBELL, M. D.
EDWARD B. ANGELL, M. D., ROCHESTER, N. Y.

SCRIPTA FERUNT ANNOS

BUFFALO:

1888.

Entered at the Post-Office at Buffalo, N. Y., as Second-class Mail Matter.

*Jno. Laughlin & Co., Printers and Binders, Buffalo, N. Y.*

